# Targeting Virulence Factors of *Candida albicans* with Natural Products

**DOI:** 10.3390/foods11192951

**Published:** 2022-09-21

**Authors:** Qing-Ru Bu, Meng-Yuan Bao, Yue Yang, Tian-Ming Wang, Chang-Zhong Wang

**Affiliations:** 1 School of Pharmacy, Anhui University of Chinese Medicine, 350 Longzihu Road, Hefei 230012, China; 2 Institute of Integrated Chinese and Western Medicine, Anhui Academy of Chinese Medicine, Hefei 230012, China; 3 Department of Microbiology and Immunology, School of Integrated Chinese and Western Medicine, Anhui University of Chinese Medicine, 350 Longzihu Road, Hefei 230012, China

**Keywords:** agri-foods, functional compounds, natural products, *Candida albicans*, virulence factor

## Abstract

Natural products derived from natural resources, including nutritional functional food, play an important role in human health. In recent years, the study of anti-fungal and other properties of agri-foods and derived functional compounds has been a hot research topic. *Candida albicans* is a parasitic fungus that thrives on human mucosal surfaces, which are colonized through opportunistic infection. It is the most prevalent cause of invasive fungal infection in immunocompromised individuals, resulting in a wide variety of clinical symptoms. Moreover, the efficacy of classical therapeutic medications such as fluconazole is often limited by the development of resistance. There is an ongoing need for the development of novel and effective antifungal therapy and medications. Infection of *C. albicans* is influenced by a great quantity of virulence factors, like adhesion, invasion-promoting enzymes, mycelial growth, and phenotypic change, and among others. Furthermore, various natural products especially from food sources that target *C. albicans* virulence factors have been researched, providing promising prospects for *C. albicans* prevention and treatment. In this review, we discuss the virulence factors of *C. albicans* and how functional foods and derived functional compounds affect them. Our hope is that this review will stimulate additional thoughts and suggestions regarding nutritional functional food and therapeutic development for patients afflicted with *C. albicans*.

## 1. Introduction

Agri-foods and derived components are important constituents of natural products. Natural products serve as an essential source of drug development due to their remarkable pharmacological activity, good compatibility, and few side effects compared to synthetic compounds. Functional compounds, which specific reference to plant-derived active ingredients with promising pharmacological activity, attracted the attention of researchers all over the world. There is more and more proof of the possible protective effects of particular foods and food bioactive ingredients that are relevant to human health. It is necessary for researchers to explore the connection between the biological activity of natural products and relevant mechanisms, and their application in the field of nutritional functional foods as well as in the field of life and health.

Candidiasis caused by *Candida* spp. affects more than 400,000 people each year, with a 50% fatality rate among these individuals [1,2]. Invasive candidiasis is the most common deep-seated fungal disease in critically ill patients. The incidence rate is approximately 2.6–16.5%, and the mortality rate can be as high as 40–60% [3,4]. *Candida albicans* is the most opportunistic pathogenic *Candida* species that causes infections may range, in scale, from affecting the mucous membranes to being systemic infections [5]. *C. albicans* infection rates have risen substantially in recent years, particularly for systemic infection, due to the widespread use of antibiotics and immunosuppressants, the use of radiotherapy and chemotherapy in tumor patients, and the spread of the immunodeficiency virus [6,7]. Even with sufficient antifungal treatment, patients have a death rate of up to 40% [8]. Generally, *C. albicans* is able to colonise the skin and mucosal membrane surfaces of most healthy individuals [9]. When the normal defense function of the body is damaged, *C. albicans* can progress from being a superficial mucosal infection in the mouth, throat, and reproductive tract to a systemic invasive candidiasis affecting the circulatory system, bone, and brain [7]. *C. albicans* pathogenicity is associated with the virulence factors: morphological transition between yeast and hyphae, the production of adhesions and invasins, biofilm formation, phenotypic transformation, and hydrolases secretion [10].

**Figure 1:**
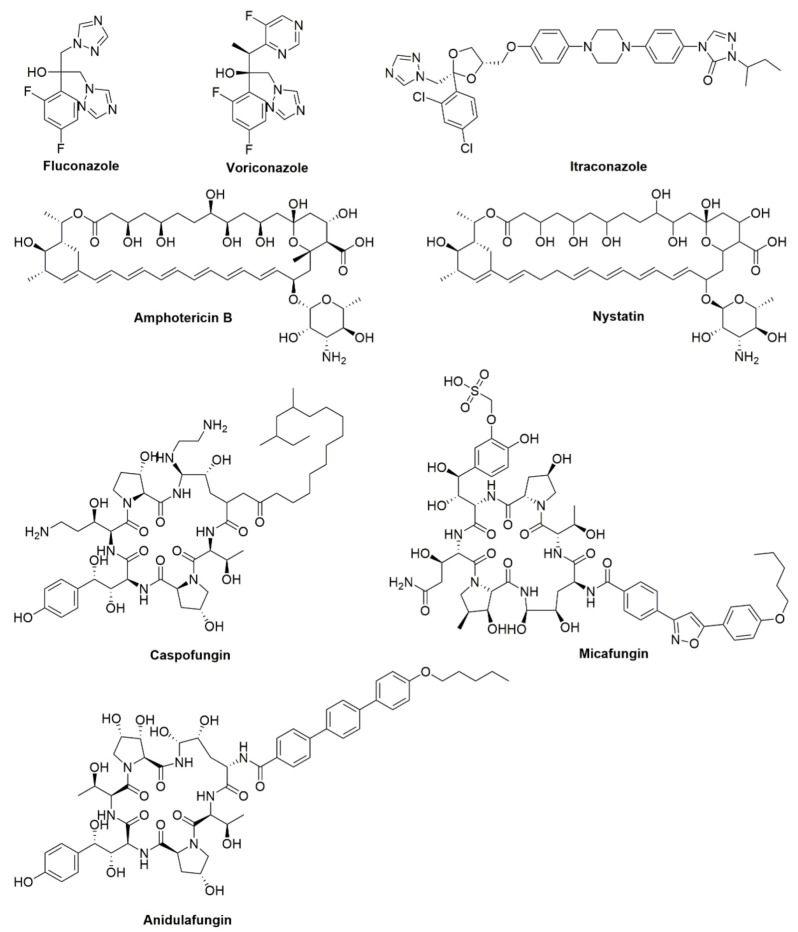
Chemical structures of traditional antifungal agents acting on *C. albicans*.

Clinically, candidiasis treatment medications are classified into three categories of polyenes, azoles, and echinocandins, they both target ergosterol and 1,3-D-glucan synthase located on the fungal cell membrane and fungal cell wall respectively [11] (Figure 1). Thompson et al. summarized, in detail, the effects of related traditional antifungal agents on *C. albicans* (Table 1) [11]. Polyene drugs such as nystatin and amphotericin B (AmB) are commonly used. However, dose-limiting toxicities, drug interactions, the development of drug resistance, and other factors limit the use of these agents. For example, nystatin, which is mainly used to treat the overgrowth of *Candida* spp., especially *C. albicans*, is limited to topical use because of insignificant oral absorption and systemic toxicity from intravenous administration [12,13,14]. AmB is an effective drug with the widest antibacterial spectrum for deep fungal infection. However, the side effects and significant toxicity of AmB have limited its clinical use in oral candidiasis [15]. Triazoles, which include fluconazole, are currently the most widely used antifungal drugs. Fluconazole (FLZ) is the first choice in the early stage of clinical treatment of *Candida* spp. infection. It remains at a subtherapeutic level in the human body for long periods, leading to increased drug resistance [16]. Major triazoles also include and voriconazole and itraconazole. With long-term drug abuse and due to unnecessary use in combined medication, *C. albicans* resistance to oxazole drugs is increasing, including through cross-resistance to a variety of oxazole derivatives, limiting treatment options for patients with oxazole-resistant *Candida* spp. Echinocandins, such as caspofungin, mycamine, and anidulafungin, are another class of antifungal drugs. These antifungal drugs have limited clinical use due to the high incidence rate and mortality associated with *Candida* spp. as well as the emergence of drug resistance and side effects.

**Figure 2:**
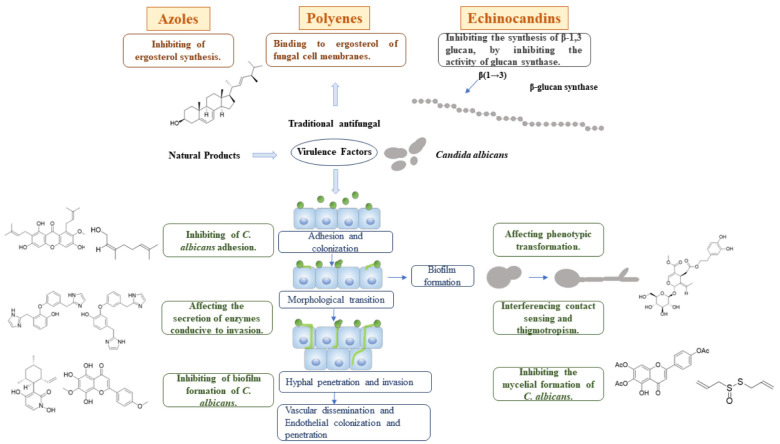
Regulation of traditional antifungal drugs and natural products on virulence factors of *C. albicans*.

**Figure 3:**
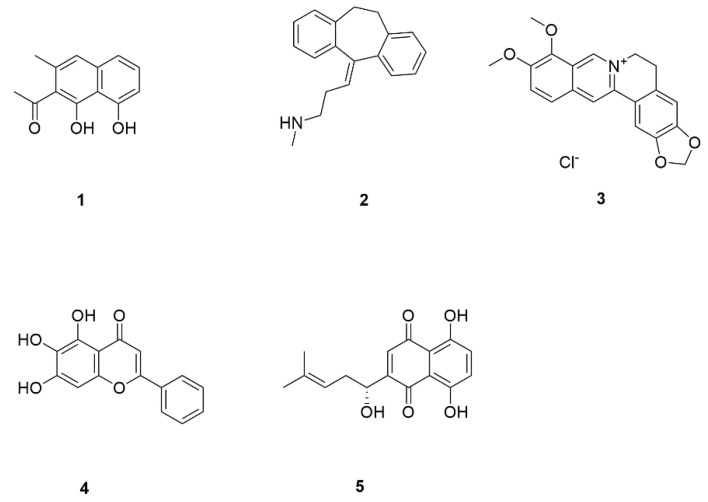
Chemical structures of natural compounds targeting various virulence factors.

There is an urgent need to develop new antifungal drug candidates and therapeutic methods [17,18]. During the last two decades, the situation has been exacerbated due to lack of research to develop antifungal agents with novel mechanisms of action [19]. Compared with targeting the growth process of fungal cells, targeting virulence is a better choice for the development of new antifungal drugs. Targeting *C. albicans* virulence factors helps us to mine or design highly specific antifungal drugs that avoid or minimize side effects on the host. The discovery of virulence factors can significantly increase the number of potential targets for antifungal drug development, as well as lead to new therapeutic categories with novel mechanisms [20]. In recent years, a number of natural functional compounds and botanical preparations (Table 2) were found to be effective against the virulence factors of *C. albicans* (Figure 2). It has been reported that nepodin (**1**, Figure 3), a seasoning, derived from *Rumex japonicus* roots, effectively inhibits *C. albicans* biofilm formation without affecting the growth of its planktonic cells [21].

Line (NOR) (**2**, Figure 3) can inhibit the formation of biofilm and hyphae and effectively kill cells within mature biofilms [22,23,24]. Moreover, natural products such as berberine (**3**, Figure 3) from bayberry [25], skullcap (**4**, Figure 3) [27], and shikonin (**5**, Figure 3) [28] can be used in conjunction with FLZ to reduce the dose of FLZ. In the field of *C. albicans* research, finding and developing new antifungal medications is still a difficult job. This paper aims to elaborate on *C. albicans* virulence factors and the effect of clinical drugs and natural product intervention on *C. albicans* virulence factors, which is expected to provide new ideas for *C. albicans* prevention and treatment.

## 2. Virulence Factors in *C. albicans* and Natural Products with Regulating Activity

*C. albicans* predominates on the host gastrointestinal mucosa. It can infect the blood and deep tissues when the host immune system is weak, giving it an aggressive nature [65]. Its invasion of the host process is divided into key steps of adhesion and colonization, hyphal invasion, and immune escape. Firstly, it must adhere to organisms or various nonbiological materials in vivo (such as dentures, urinary catheters, etc.) to form a biofilm structure wrapped by single-layer or multi-layer cells and their secreted extracellular polymeric matrix. Adhesion contributes to the persistence of organisms in the host, which is very important for the spread of fungi. Adhesion of *C. albicans* to host cells is the first and necessary step of infection and an important prerequisite for invasion. Many biomolecules on the surface of bacteria increase their adherence to host cells and are referred to as “adhesin” [66].

### 2.1. Adhesion

#### 2.1.1. Adhesin

The best researched of adhesins are the agglutinin-like sequence proteins (Als) and Hwp1 [67]. Proteins in the lectin-like sequence family of *C. albicans* are among the most characterized fungal adhesins [68]. In this species, the Als family contains eight genes encoding large cell surface glycoproteins. These glycoproteins have similar basic structures, consisting of an N-terminal domain with adhesion function (MT-Als), a central structure of tandem repeats, and a C-terminal domain rich in Ser/Thr. There is a secretory signal sequence at the N-terminal of the protein and a glycosylphosphatidylinositol (GPI) anchored addition site at the C-terminal, which is consistent with the protein entering the secretory pathway and its final localization to the fungal cell wall β-1,6-glucan [69]. *Als* gene can encode GPI-like cell surface glycoprotein, and APX001A is an inhibitor of GPI anchor protein. It inhibits the synthesis of GPI by inhibiting the inositol acylation reaction of glucosamine-PI and reduces the content of GPI anchor protein on the cell surface to inhibit the life processes related to fungal virulence, such as fungal adhesion, mycelial growth, and biofilm formation. At present, the phase I clinical study has been completed, and the phase II clinical study is recruiting patients for the first-line treatment of candidemia [70].

Another important *C. albicans* adhesin is Hwp1, which is also related to the mycelial associated protein GPI-like protein. Hwp1 is a cell surface protein of *C. albicans* with characteristics useful for infection. It means a lot to hyphal formation and yeast adhesion to epithelial cells. Hwp1 is a substrate of mammalian transglutaminase, which can bind *C. albicans* hyphae and host cells through covalent links, resulting in *C. albicans* infection [71]. *Hwp1* gene encodes *C. albicans* proteins involved in a variety of functions, including cell wall assembly, intracellular signal transduction, and hyphal development. In addition, it can promote the combination of *Candida* spp. and epithelial cells as the first step of colonization. *C. albicans* with the *Hwp1* gene deleted cannot form stable covalent bond mediated adhesions with human oral epithelial cells, which indicates that *Hwp1* plays an important role in the pathogenesis of *C. albicans*, making Hwp1 is a potential drug target [72].

#### 2.1.2. Invasin

*C. albicans* can invade host cells through two different mechanisms: inducible endocytosis and active infiltration. Inducible endocytosis, which is the most researched, refers to the process by which fungi express a special protein, invasion, on the cell surface (like E-cadherin of epithelial cells) that binds to host ligands to induce swallowing of fungal cells as a means for them to enter host cells [73]. One mechanism by which *C. albicans* hyphae invade oral epithelium is to stimulate endocytosis by expressing Als3 and Ssa1 invasins and interacting with epidermal growth factor receptor (EGFR) on epithelial cells [74].

Als3p is the most studied among the proteins encoded by *Als3* and is among the important factors involved in the pathogenic process of *C. albicans*. Its main function is to help *C. albicans* in host colonization. For an adhesin, Als3p has extensive substrate specificity and can mediate the attachment of *C. albicans* to mountains of host cells, like epithelial cells, endothelial cells, and so on. In addition to helping *C. albicans* colonize the host, Als3p is also necessary for fungi to invade the host. In recent years, it has become a particular target of vaccines and antibodies against *C. albicans* [75,76]. Als3p-specific antibodies contain monoclonal antibodies (MAb) 3-A5, MAb113, and scFv3 as well as MAbC7, MAb3D9.3, MAb2G8, etc. An NDV-3 vaccine targeting the Als3p N-terminal has entered clinical trials. These vaccines and antibodies are expected to become efficient new antifungal drugs in the future [75,76,77].

Ssa1 is a member of the heat shock protein 70 (Hsp70) family expressed on the cell surface. It is conveyed on the surface of *C. albicans* and undertakes an invasin upon the *C. albicans* [78]. Jianning reported that the pivotal role of Ssa1 in host cell invasion is reflected in the declined ability of *Ssa1* null mutants to induce in vitro uptake by epithelial and endothelial cells themselves, and in mouse models of oropharyngeal candidiasis and disseminated candidiasis, where the virulence of the mutants was obviously diminished [78]. In addition, mutation of *Als3* and *Ssa1* mutants can reduce the expression of adhesin and invasin in epithelial cells and reduce the toxicity of *C. albicans* in a mouse model of oropharyngeal candidiasis.

**Figure 4:**
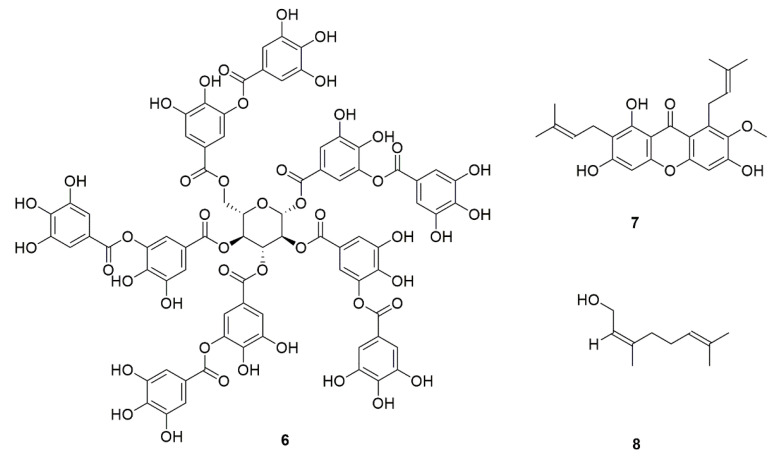
Chemical structures of natural compounds targeting adhesion.

Natural products of fruit origin can inhibit *C. albicans* adhesion, such as tannins (**6**, Figure 4) from blueberries and grapes [29], α-mangostin (**7**, Figure 4) from mangosteen [30], and raspberry extracts [31]. Nerol (**8**, Figure 4), a natural monoterpene compound, from the sweet orange of the *Rutaceae* family, was confirmed as a potential antifungal drug for the treatment of *C. albicans* invasion [32]. Furthermore, Pulsatilla decoction, a classical prescription in traditional Chinese medicine (TCM), was reported to have inhibitory effects on *C. albicans* adhesi.

### 2.2. Invasive Enzymes

*C. albicans* can secrete a variety of proteolytic enzymes conducive to invasions, such as secretory aspartate proteases (Saps) and phospholipase (PL). These are two families of extracellular *C. albicans* enzymes, some of which are related to virulence. The combined effect of Saps and PL causes yeast mycelial phase transition and adhesion to damage the host mucosa, promoting organism invasion into epithelial cells [79,80].

#### 2.2.1. Secretory Aspartate Protease (Sap)

The Sap family is thought to have 10 members, each encoding Sap1–Sap10 protein. *Sap*1–8 genes encode secretory proteases, and *Sap*9 and *Sap*10 genes encode membrane-anchored proteases [81,82]. Studies have shown that Sap is one of the important virulence factors of *C. albicans* and is essential for *C. albicans* adhesion, invasion, and pathogenicity. The expression of *Sap*4 and *Sap*5 is related to the mycelial formation, which can promote the hyphal formation. *Sap*9 and *Sap*10 enzymes enhance biofilm formation and are involved in the maintenance of cell surface integrity in *Candida* spp. [83]. Since *C. albicans* often exist in the shape of biofilms, the expression of Saps result in the formation of *C. albicans* biofilms and increases their pathogenicity. The diversity of Saps in host tissues allows for the use of different nitrogen sources in host development [84]. Therefore, the existence and expression of the *Sap* gene family endow *Candida* spp. with some adaptive advantages, especially under the selective pressure of antifungal compounds. Biofilm-associated *C. albicans* shows reduced sensitivity to both certain antifungal drugs and the killing effect of the host immune system [85]. Kumar et al. found that disruption of the gene encoding Saps reduces the ability of *C. albicans* to damage vaginal and oral epithelial cells, resulting reduced host infection [86].

Studies have shown that *Sap*2 activity is stronger in itraconazole-resistant than in itraconazole-sensitive strains, suggesting that *Sap*2 may help to improve the virulence and pathogenicity of itraconazole-resistant *C. albicans* strains [87]. According to the survey, many people with candidiasis have the habit of smoking. Alanazi et al. conducted an interesting experiment to explore whether smoking affected candidiasis. The results showed that both nicotine-free and nicotine rich e-cigarettes increased the expression of different *Sap* genes, including *Sap*2, *Sap*3, and *Sap*9, which bring about the growth and virulence of *C. albicans*. Furthermore, e-cigarettes with or without nicotine increased the growth and mycelial length of *C. albicans*. Exposure to e-cigarettes attributes to the overgrowth and virulence gene expression of *C. albicans*, which may lead to oral candidiasis in individuals carrying and using e-cigarettes [88].

#### 2.2.2. Phospholipase (PL)

The research on the pathogenicity of PL in *C. albicans* has increased in recent years. It has been confirmed that PL plays an important role in the pathogenic process. PL produced by *C. albicans* can increase the permeability and damage the integrity of the cell membrane through the decomposition of host cell membrane phospholipids, which then promotes the invasion of *C. albicans*. The PL family includes different subclasses: PLA, PLB, PLC, and PLD [89]. PLB is a secretory glycoprotein with hydrolase and phospholipase acylase activity, which is optimal at pH 6.0. PLB plays a role in the early stages of *C. albicans* host invasion, including in adhesion, invasion, and injury to epithelial cells. However, in the animal model of candidiasis, only PLB1 has been proved to be necessary for virulence [90]. Studies have shown that the virulence of *C. albicans* is significantly weakened by ring breaking of the PLB1 gene and restored through its reintroduction [91,92]. Thus, PLB1 plays a key role in host cell adhesion and invasion. In a mouse model of systemic infection, *PLB1* and *PLB5* mutations have been shown to attenuate *C. albicans* toxicity [93].

**Figure 5:**
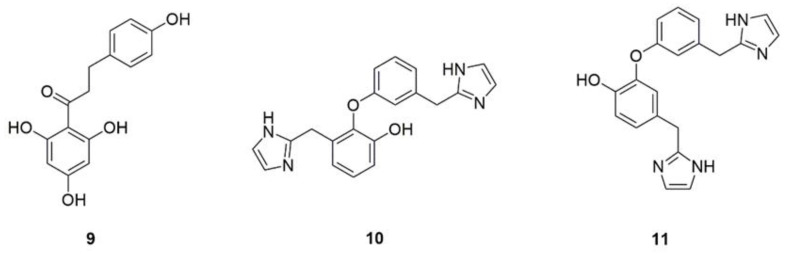
Chemical structures of natural compounds targeting invasive enzymes.

Phloretin (**9**, Figure 5) is a dihydrochalcone flavonoid derived from apples, pears and strawberries and is famous for its powerful antioxidant, anti-cancer, and anti-inflammatory properties [34,35,36]. Phloretin can suppress pathogenicity and virulence factors of *C. albicans* both in vivo and in vitro [94]. Shim et al. found that phloretin comes out antifungal activity confront some plant pathogenic fungi in vitro [95]. Phloretin shows the minimum inhibitory concentration (MIC) against *C. albicans* is 74.55 μg/mL [94]. Na Liu et al. reported that phloretin exerts through inhibition biofilm formation and inhibition of yeast hyphae transition by downregulation of hypha-associated genes, including enhanced adherence to polystyrene 1, the extent of cell elongation gene 1, *Hwp1*, and *Als3*. Phloretin represses the secretion of proteases and phospholipases by decreasing the expression of protease-encoding genes *Sap*1, *Sap*2, and PLB1. Furthermore, the in vivo antifungal activity of phloretin was supported by reversal of the enhanced lesion severity, inflammatory infiltration, and the increased colony-forming unit counts caused by *C. albicans* of tongue tissues in oral candidiasis mice.

Lepidine B (**10**, Figure 5) and lepidine E (**11**, Figure 5) [37], natural products derived from edible vegetables, *Lepidium sativum* seeds, have a certain effect on phospholipase and inhibit its production. *Origanum vulgare*, a vanilla plant, is often used in cooking, including in the preparation of sauces and the seasoning of pizza in Europe and the United States. The essential oil of *O. vulgare* causes significant reductions in the production of the phospholipases by *C. albicans* strains [38]. The methanolic extract of Juglans regia root was found to severely affect *Candida* spp. growth and hydrolytic enzyme secretions [39]. An acetone/water (7:3, *v*/*v*) crude extract of *Eugenia uniflora*, an ornamental and edible fruit in Brazil, can not only impair the secretion of phospholipase and proteases but also affect the transition from yeast to hyphae [40].

### 2.3. Biofilm Formation

Ergosterol is a specific component of fungal biofilms, a feature regarded as a critical factor in the high level of resistance of *Candida* spp. to conventional antimycotic agents [96] Therefore, ergosterol is often used as a target for azoles. Biofilm formation is a continuous process, including yeast cell adhesion to the substrate, yeast cell proliferation, mycelial cell formation on biofilm, accumulation of extracellular matrix materials, and the final dissemination of yeast cells from biofilm [97]. Drug resistance caused by the biofilm formation of *C. albicans* is among the most vital reasons for the failure of antifungal therapy.

Azole antifungals cause drug resistance by affecting ergosterol production and gene and protein expression [98]. Studies have found that Hwp1 and Als3 are connection with the formation of *C. albicans* biofilm. Deng et al. discussed that the *Als3* gene is differentially expressed in suspended antifungal drug-sensitive *C. albicans* cells. Expression of the *Als3* gene was higher in *C. albicans* with biofilm formation than without. The study also confirmed that the high *Als3* gene expression group had a higher rate of biofilm development than the low *Als3* gene expression group [99]. Deng et al. pointed out that *C. albicans* with biofilm formation had stronger resistance to FLZ, voriconazole, and itraconazole but maintained sensitivity to caspofungin (CAS) and micafungin in vitro and in vivo [99]. It can be seen that biofilm formation, as a virulence factor of *C. albicans*, is a very important determinant for the drug resistance of *C. albicans*. In-depth research on *C. albicans* biofilm formation will help to guide the prevention and treatment using clinical antifungal agents. on the biofilm formation of *C. albicans* will help to guide the prevention and treatment.

**Figure 6:**
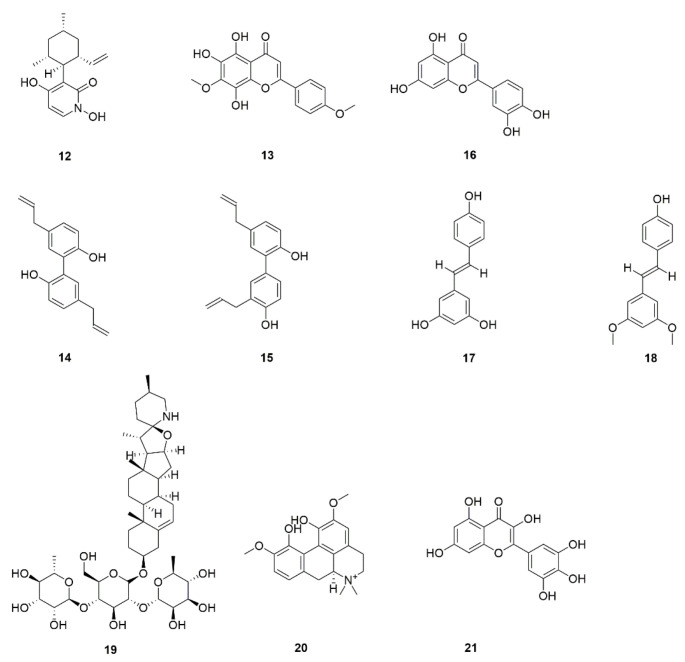
Chemical structures of natural compounds targeting biofilm formation.

*C. albicans* can rapidly develop resistance to antifungal drugs through various mechanisms, including through mutation of the *Erg11* gene involved in the ergosterol biosynthesis pathway. In addition, some studies reported amino acid substitution and frameshift mutations prevent the combination of drugs with target enzymes [100]. These mutations prevent drug binding and inactivate ergosterol [100]. Drug resistance does not affect virulence, and an increase in ergosterol does not translate into increased resistance to cell surface damaging agents because it may also be affected by other factors such as chitin and glucan concentrations [101]. FLZ resistance is linked with increased ergosterol content in the plasma membrane. AmB targets ergosterol on the cell membrane and exhibits high fungicidal activity [101]. Pyridoxine, known as vitamin B6 (**12**, Figure 6), is a small natural product isolated from fruit and endolichenic fungus [41], some are derived from vegetables. Pyridoxine has been previously reported to exhibit excellent antifungal activity against *C. albicans* by interfering with the ergosterol synthesis with its MIC of 1.6 μg/mL [102]. 5,6,8-Trihydroxy-7,4′dimethoxyflavone (**13**, Figure 6), separated from *Dodonaea viscosa* var *angustifolia*, has the ability to inhibit ergosterol synthesis and the production of hyphae and biofilm in *C. albicans* [42]. Nortriptyline (NOR) (2) belongs to the group of tricyclic drugs, which can inhibit the formation of biofilm and hyphae and can effectively kill cells in a mature biofilm of *C. albicans*. *C. albicans* GRACE^TM^ mutant and Haplo defect analysis was used to identify the potential targets of NOR and screened in parallel with AmB, CAS, and FLZ. The results showed that NOR can be used as a new antibacterial drug and has great potential to be used in an infection model in vivo. The combined application of NOR and AmB could increase the antifungal activity by 3–4 times than a single agent [22,23,24].

Magnolol (**14**, Figure 6) and honokiol (**15**, Figure 6) prevent biofilm formation in *C. albicans* by the Ras1/cAMP/Efg1 pathway [43]. Luteolin (**16**, Figure 6), an interesting naturally occurring flavonoid substance obtained from vegetables, fruits, and certain medicinal plants like *Mentha spicate* and perilla, blocks biofilm formation and inhibits the adhesion of *C. albicans* at 16 μg/mL [44]. In addition, resveratrol (**17**, Figure 6) from grapes and other foods and pterostilbene (**18**, Figure 6) from Vitis rupestris have been shown to inhibit the formation of biofilms and destroy preformed biofilms [45]. Solamargine (**19**, Figure 6), a steroidal glycoaloid derived from *Solanum mammosum*, has been shown to be the most active compound against *C. albicans* in vitro [46]. Magnoflorine (**20**, Figure 6) [47] and *Rosmarinus officinalis* extract [48] both have a pronounced antibiofilm effect. *R. officinalis*, a spice used in steaks, potatoes and other dishes, as well as grilled products. *Adenophora triphylla* var. *japonica* extract [49], lemongrass extract [50], and myricetin (**21**, Figure 6) [51] from *Solanum scabrum* can inhibit the formation of biofilms to a certain extent.

### 2.4. Phenotypic Transformation

**Figure 7:**
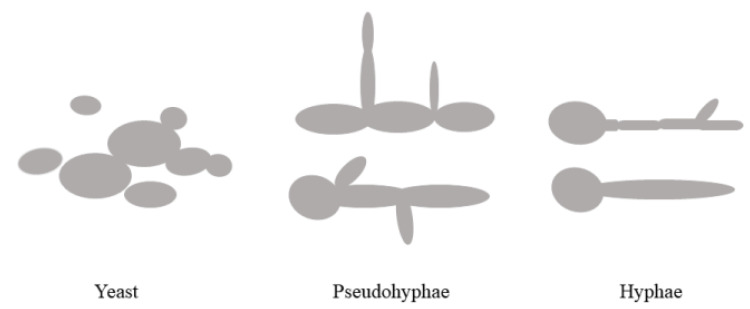
The three forms of *C. albicans*.

*C. albicans* is a single-cell, yeast-like fungus that can form biofilms with a thickness of about 25 μm. It is a biphasic fungus with morphological diversity. There are several growth forms in terms of cell shape, such as yeast phase, pseudohyphae, and hyphae (Figure 7), and different forms of cells can be converted to each other [103]. During invasion, *C. albicans* exists in the form of yeast, while during parasitic or bloodborne transmission, *C. albicans* exists in the form of mycelium. This morphological transformation is controlled by protein products of specific *C. albicans* genes, which guide spores into germ tubes or mycelium and then promote adhesion. The morphological transformation between yeast and hyphae plays an essential role in the virulence of *C. albicans* [20]. It can detect various extracellular stimuli and trigger the adaptive transition from yeast phase to mycelium phase by transmitting signal molecules in a step-by-step manner through the intracellular signal transduction system. Yeast hyphal phase morphological transformation, a typical morphological transformation system, is closely related to the adhesion and invasiveness of *C. albicans*. The ability of *C. albicans* to transition between yeast and hyphal development when stimulated by a special host environment influence its invasiveness. Experiments reveal that mycelial *C. albicans* has a better capacity for adhesion and invasion of the host and escape the host immune system response, whereas yeast *C. albicans* has little or very little pathogenicity.

At present, the consensus resulting from research on the morphological transformation of *C. albicans* is that the extensive and in-depth signaling pathways mainly include the cyclic adenosine monophosphate/protein kinase A (cAMP/PKA) signaling pathway, mitogen-activated protein kinase (MAPK) signaling pathway, Rim101-mediated pH signal pathway, and Tup1-mediated negative regulation signaling pathway [104].

The transition between yeast and invasive hyphae is central to virulence [65], phenotypic transformation is often accompanied by changes in some virulence factors, such as mycelial specific genes *Hwp1* and *Als3*, and is closely related to the hydrolysis of *C. albicans*. Because adhesion and pathogenicity are closely related, mycelia adhere to host cell surfaces more easily than yeast phase cells.

**Figure 8:**
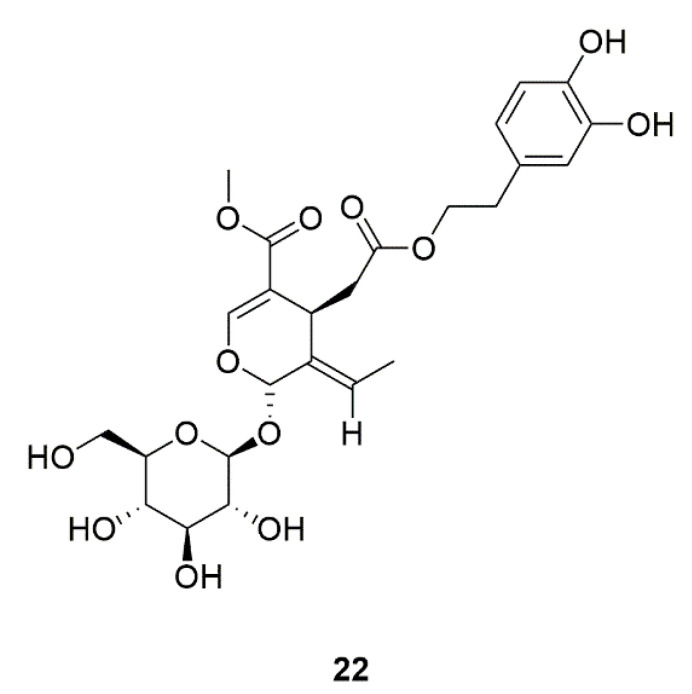
Chemical structures of natural compounds targeting phenotypic transformation.

Furthermore, some natural products such as oleuropein (**22**, Figure 8), which is derived from *Syringa reticulata*, can regulate the morphological transformation of *C. albicans* [52]. *Paeonia lactiflora* can not only used for ornamental purposes, but also used to make flower cakes or flower teas in China, Japanese and other countries. *Paeonia lactiflora* ethanol extract shows a good inhibitory effect on biofilm formation by impeding cell adhesion via downregulation of the protein expression levels of Als3, Hwp1, Sap1, and Ece1, obstructing the morphological transition from pseudohyphal to hyphal filaments [53].

### 2.5. Contact Sensing and Thigmotropism

Environmental conditions such as low oxygen environment, nutritional deficiency, and osmotic pressure changes have a very important impact on fungi growth, with contact sensing being a very important environmental signal. By contacting the material surface fungi can understand and adapt to changes in the surrounding environment, triggering the formation of *C. albicans* biofilm. In addition, contact sensing of *C. albicans* will also lead to the formation and growth of invasive hyphae, and fungi then invade human tissues and blood, grow, and reproduce, resulting in pathological changes and pathophysiological processes of tissue damage, organ dysfunction, and inflammatory response.

Thigmotropism means that human pathogenic fungi such as *C. albicans* reposition the long axis of a hypha to adapt to the potential surface morphology. Although most of the hyphae of *C. albicans* in tissues are randomly distributed, many in vivo experiments have shown that hyphae are distributed along or perpendicular to the stratum corneum in the epidermal keratosis layer. This contact sensing of hyphae determines the arrangement of hyphae on the stratum corneum microsurface, and it has also been determined that they more easily adhere to host cells compared with yeast type cells. Brand et al. found that the nematicity of *C. albicans* hyphae is controled by calcium channel proteins Cch1 and Mid1 [105], which is closely correlated with the morphology, environmental stress responses, and pathogenicity of *C. albicans*. Hyphae can find the damaged surface of epithelium and endothelium and penetrate the host tissue due to contact orientation. Although contact sensing and thigmotropism are not the main virulence factors of *C. albicans* and there are relatively few studies on contact sensing and thigmotropism, they actually have a greater impact on the growth of fungi and deserve further investigation.

### 2.6. Signaling Pathways Related to Mycelial Formation

The fungus *C. albicans*, like all living organisms, is constantly responding to changes in the environmental conditions. Signaling pathways, which generate appropriate intracellular signaling activity by sensing external changes, lead to genetic and physiological changes, complete cellular responses, and thus adapt to environmental changes. Multiple stimuli that act through multiple complex signal transduction pathways can trigger hyphal formation [20]. The hyphal form is the pathogenic form of *C. albicans*. At present, it has been confirmed that there are several phenotypic conversion signal transduction pathways in *C. albicans* that respond to different environmental signals. The most common two are the MAPK pathway [106,107,108] and the cAMP/PKA pathway [109,110].

#### 2.6.1. MAPK Signal Pathway

MAPK pathways are important pathways in eukaryotic signal transduction networks. The MAPK signaling pathway includes three kinases, namely MAP kinase (MAPK), MAPK kinase (MEK), and MEK kinase (MKKK) [111]. After the cells are stimulated, MAPK is activated through the progressive phosphorylation of MKK and MKKK. Four different MAPK signal transduction pathways have been found in mammalian cells: the ERK1/2 pathway regulates cell growth and differentiation, the JNK and p38 MAPK pathway play an important role in stress responses such as inflammation and apoptosis, and the ERK5 pathway is involved in angiogenesis [111]. MAPK signaling pathways widely exist in many immune cells, such as macrophages, dendritic cells, neutrophils, T cells, and B cells. As an important signaling pathway in cell defense systems, the MAPK signaling pathway is an important target for bacterial pathogen destruction [112].

Toenjes et al. screened five small molecules and found that these compounds can inhibit the transformation of *C. albicans* yeast to mycelium in response to carbon limitation [113,114]. These known compounds are inhibitors of protein kinase, protein phosphatase, Ras signaling pathway, G protein-coupled receptor, calcium homeostasis, nitric oxide, and guanylate cyclase signaling, and apoptosis in mammalian cells [20].

#### 2.6.2. cAMP/PKA Signaling Pathway

**Figure 9:**
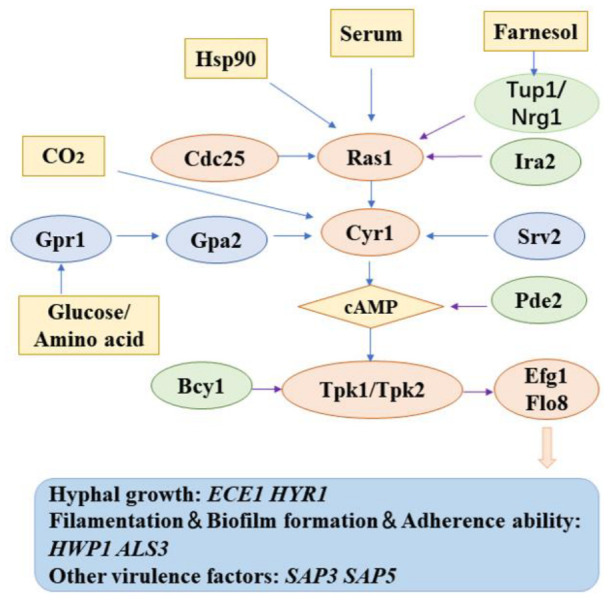
Schematic diagram of Ras/cAMP/PKA pathway in *C. albicans* (blue arrow represents activation and purple arrow represents inhibition).

Hyphal formation, morphological transformation, biofilm formation, sterol synthesis, glycolysis, and other biological and metabolic processes are very important for the growth, reproduction, and pathogenicity of *C. albicans* [115,116]. These processes are regulated by multiple signal pathways, of which the cAMP/PKA pathway (Figure 9) is a widely studied and widely used pathway in the regulation of morphological transformation [117]. It plays a key regulatory role in the process of *C. albicans* morphological transformation. Ras protein is a highly conserved GTPase (small GTPase) protein 1 in eukaryotes, which is located upstream of the Ras/cAMP/PKA signal transduction pathway [118]. Ras, which belongs to the small G protein family has two forms: GTP binding activated state and GDP binding inactive state. When it is in the activated state, it can activate downstream effector molecules. The conversion between the two depends on the GTPase activity of Ras itself [119].

*C. albicans* contains two Ras proteins called Ras1 and Ras2. Ras1 protein is necessary for *C. albicans* mycelial growth and virulence. It was found that the mycelial development defect and virulence of the strains with *Ras1* gene knockout were significantly reduced [120]. It was further supplemented with cAMP or protein kinase components in the overexpression MAPK pathway. It was found that the mycelial development defect of the strains with *Ras1* gene deletion could be reversed, which confirms that Ras1 regulates the downstream cAMP/PKA and MAPK signal transduction pathway to complete cell signal transduction [121]. In addition, the latest research shows that in *Ras1*-deficient bacteria, there is a reduction in ribosomal biosynthesis mediated by Torc1, resulting in the increased tolerance of the strain to AmB [122].

cAMP is an important second messenger molecule in organisms. cAMP activates PKA (cAMP-dependent protein kinase) to phosphorylate the target protein and produce subsequent effects. Finally, cAMP is hydrolyzed into 5′-AMP by phosphodiesterase (Pde), including Pde2, and inactivated. During the budding process of *C. albicans*, the high expression of Pde2 can antagonize the cAMP synthesis activated by Srv2 and inhibit the formation of mycelium. In bacteria with *Pde2* deletion, high levels of cAMP and extraordinary growth of mycelium were observed, but the state of extraordinary mycelium growth and nonmycelial state both lacked virulence [123]. Moreover, the study confirmed that the intracellular cAMP levels of the *Pde2* gene knockout strain were significantly increased, and the constitutively activated cAMP signaling pathway regulates filamentous mycelial growth and toxicity [124]. Deficiency of *Pde2* leads to reduce mycelial growth and virulence in a mouse model of systemic infection [125].

PKA is also known as cAMP-dependent protein kinase A, since its activation is dependent on cAMP. When its levels increase, cAMP binds to the regulatory subunit of PKA to change the conformation and release the catalytic subunit to activate PKA. The regulatory subunit of PKA in *C. albicans* is encoded by *Bcy1*, and the catalytic subunit is encoded by *Tpk 1* and *Tpk 2*, respectively [126].

Efg1 (enhanced filamentous growth 1) is a class of APSES proteins that mediate cAMP/PKA signal transduction pathway encoded by *Efg1* (enhanced filamentous growth). Efg1, as a positive regulator of yeast mycelial morphogenesis, is mostly relies on the Ras/cAMP/PKA signal pathway [127].

**Figure 10:**
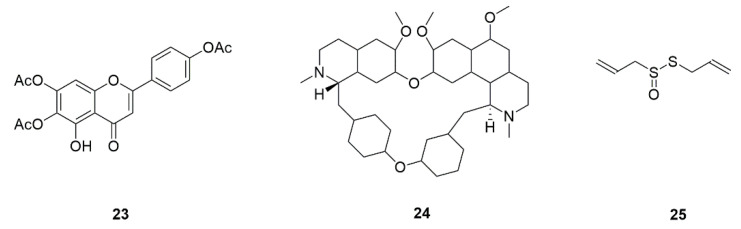
Chemical structures of natural compounds targeting mycelial formation.

Studies have shown that *Efg1* gene knockout strains have defective mycelial formation and decreased mycelial specific gene expression under the action of most mycelium-inducing factors such as serum, indicating that Efg1 plays a very important part in regulating the mycelial growth of *C. albicans* [128]. The combination of 6,7,4′-O-triacetylxanthin (TA) (**23**, Figure 10) and FLZ had a strong synergistic inhibitory effect on the biofilm formation of drug-resistant *C. albicans* [54]. TA combined with FLZ extended the survival rate and reduced tissue invasion in mice infected *C. albicans*. The combination of TA and FLZ also strongly prevented the yeast mycelial transformation of *C. albicans* and immensely decreased the expression of Ras/cAMP/PKA signal pathway concerned genes (*Ras1* and *Efg1*) and mycelial related genes (*Hwp1* and *Ece1*). The results showed that TA allianced with FLZ inhibited hyphal and biofilm-formation by Ras/cAMP/PKA signal pathway, thereby reducing the infectivity and drug resistance of *C. albicans* [54].

Tetrandrine (**24**, Figure 10), which is a bis-benzylisoquinoline alkaloid compound extracted from several natural plant sources, covering Stephania tetrandra [55,56]. Tetrandrine curbs biofilm formation by diminishing adhesion and morphological transition, instead of inhibiting the growth of *C. albicans*. The mechanism of anti-biofilm may be in connection with the Ras/cAMP pathway [129]. One study confirmed that a compound from garlic extract, allicin (**25**, Figure 10), was also able to suppress hyphae formation in *C. albicans* [57].

## 3. Other Drugs

**Figure 11:**
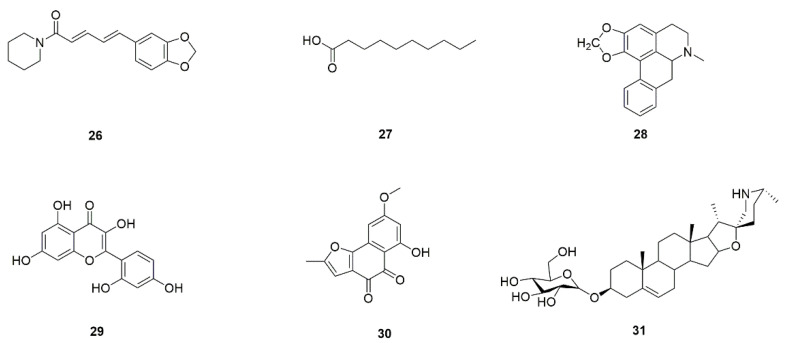
Chemical structures of natural compounds targeting multiple virulence factors.

In addition to FLZ, CAS, AmB and other drugs commonly used for the cure of candidiasis, studies in recent years have shown that a variety of drugs, including some natural products, have a certain effect on *C. albicans* infection. Some natural products can act not only on a single virulence factor but also on several virulence factors of *C. albicans* (Figure 11).

Studies have shown that piperine (**26**, Figure 11) from pepper can regulate the morphological transformation between yeast and mycelium by inhibiting mycelial extension and converting from the mycelial phase into the yeast form without affecting the host [58]. Piperine markedly reduces the biofilm formation of *C. albicans* at at 32 μg/mL without influencing the normal cellular and metabolic viability. Additionally, microscopic analysis demonstrated that piperine effectively inhibited adherence of *C. albicans* to surfaces as well as restricts the formation of hyphal in a dose-dependent manner [58]. It can be seen that piperine has significant effects on a variety of virulence factors of *C. albicans* and could be regarded as a candidate drug for the treatment of biofilm-related *C. albicans* infection [58]. *Padma Hepaten* from amla fruit and green tea from *Camellia sinensis* administration in a dose-dependent manner, and synergistically inhibits the growth of *C. albicans* biofilm in vitro, its ability to secrete exopolysaccharides, as well as the transformation of yeast into hypha that is essential for the fungus virulence. Additionally, it also has an influence on the expression of the *Hwp1* and *Als3* virulence-linked genes. Consequently, *Padma Hepaten* and green tea may contribute to the fight against of the *C. albicans* infections and raising drug resistance [59].

Recently, Kumar et al. found that decanoic acid (**27**, Figure 11) from animal fat can effectively inhibit the transformation from yeast to hyphae, adhesion, and biofilm formation of *C. albicans* but without hindering fungal growth. Gene expression analysis suggests that decanoic acid may function by inhibiting Hwp1 and Efg1 as analogs of farnirol, a known biofilm inhibitor [60]. In brief, decanoic acid inhibits the expression of Efg1, which is a positive regulator of Hwp1 in *C. albicans*.

Roemerine (**28**, Figure 11) is derived from the fresh rattan stem of *Fibraurea recisa* and some from the lotus leaf, which is used as beauty-slimming tea in our everyday life. Roemerine can inhibit the yeast-to-hyphae transition of *C. albicans* in a dose-dependent manner and significantly inhibits the biofilm formation. The anti-biofilm mechanism may be related to the cAMP pathway [61]. Morin (**29**, Figure 11), a flavonoid, which is found in several medicinal plants, consisting of *Maclura tinctoria*, *Maclura pomifera*, and *Psidium guajava*, that manifest extensive biological properties [62]. Morin treatment remarkably inhibits the formation of *C. albicans* biofilm in a concentration-dependent manner. Besides, the production of virulence factors, covering hyphal formation, phospholipase, protease and invasion, were also significantly attenuated upon treatment with morin at its minimum biofilm inhibitory concentration (MBIC) [130].

Berberine hydrochloride (BH, **3**), an active constituent of Coptis chinensis and other plants, has a wide range of antibacterial activities and can be used to treat Candida infection. BH can inhibit the formation of germ tubes and hyphae, by regulating the MAPK pathway, and increase exposure of chitin and β-1,3-glucan. Especially, the upregulation of the core genes *Sln1*, *Ssk2*, *Hog1*, and *Pbs2* may make a difference in the expression of key downstream factors correlated with germ tube and hyphal formation (*Hwp1*) and cell wall integrity (*Chs3* and *Gsc1*). BH affects a quantity of biological processes in *C. albicans*, and may therefore be an valid substitute to traditional azole antifungal agents [26].

Biatriosporin D (**30**, Figure 11), isolated from the endolichenic fungus *Biatriospora* spp., displays antivirulence activity by inhibiting the adhesion, hyphal morphogenesis, and biofilm formation of *C. albicans* [63].

*Solanum nigrum*, a medicinal and edible plant, is edible in both berries and leaves. Solasodine-3-O-β-D-glucopyranoside (SG, **31**, Figure 11), a steroidal alkaloid glycoside, separated from *S. nigrum*, which attenuates the virulence of *C. albicans* by inhibiting its adhesion and the morphological transition. Additionally, SG observably subdues biofilm formation and has killing activity against mature biofilm. Further research has shown that inhibiting the Ras/cAMP/PKA signaling pathway and reducing the cAMP contents can effectively reduce its bioactivity [64].

## 4. Perspectives and Conclusions

Natural products and their relevanted components have historically and regionally been used to remedy of a good supply of diseases. Many medicinal and food homologous natural products, such as fruits, vegetables, nuts, cooking spices and other agri-foods have promising antifungal and other pharmacological activities. Nevertheless, in certain circumstances, there is still a lack of scientific proof for these natural products that have been used.

With the popularization and application of traditional single treatment drugs, people are becoming increasingly inclined to develop new *C. albicans* treatment drugs or treatment methods. Morphological conversion and hyphal formation are the two most important virulence factors of *C. albicans*, and the research on natural products targeting these factors is also the most extensive area of related research. However, due to the concept of targeted therapy not long after the concept was proposed, most related drug research and development has not officially entered the clinical stages, so the demand for traditional antifungal drugs is still very high, and the development of targeted drugs is very urgent. Besides, extracting pure compounds from natural sources is complicated and costly. It is important and necessary to encourage organic chemists to find a synthetic strategy that avoids cumbersome purification steps to obtain pure compounds. Certain virulence factors, such as contact sensing and thigmotropism, have a huge space for exploration as drug targets.

With the expansion of the population infected by *Candida* spp. and the enhancement of drug resistance, targeting *C. albicans* virulence factor is a new research direction in the fight against *Candida* spp., and at the same time, the research and development of natural functional compounds especially natural products from food sources in the field of candidiasis treatment is also expanding. They have a wide range of pharmacological properties and fewer side effects and becoming a valuable research field [131,132]. Two-drugs or multi-drug combination in the treatment process of *C. albicans* has also provided surprises for the further development of antibacterial drugs, laying the foundation for the clinical application of virulence factors as potential drug targets. The study of virulence factors as potential drug targets have great influence on the treatment of *C. albicans* infection, and more in-depth studies of the associated mechanisms of action and internal relationships need to be further studied.

## Figures and Tables

**Table 1:** The effects of traditional antifungal agents on *C. albicans*.

Traditional Antifungal Agent	Type	Function
Fluconazole	Azoles	Inhibit cytochrome P450 (CYP)-dependent 14-α-demethylase and prevent the conversion of lanosterol to ergosterol.
Voriconazole		
Itraconazole		
Amphotericin B	Polyenes	Bind to ergosterol of fungal cell membranes.
Nystatin		
Caspofungin	Echinocandins	Inhibit the synthesis of β-1,3 glucan, by inhibiting the activity of glucan synthase.
Micafungin		
Anidulafungin		

**Table 2:** The effects of natural products on *C. albicans* virulence factors.

No.	Natural Products	Source	Function	Ref.
1	Nepodin (**1**)	*Rumex japonicus*	Inhibits *C. albicans* biofilm formation.	[21]
2	Nortriptyline (**2**)	Metabolites of amitriptyline	Inhibits the formation of biofilm and hyphae and effectively kills cells in mature biofilm.	[22,23,24]
3	Berberine (**3**)	Bayberry, *Coptis chinensis*	Inhibits the formation of germ tubes and hyphae by regulating the MAPK pathway and increasing exposure of chitin and β-1,3-glucan.	[25,26]
4	Skullcap (**4**)	*Scutellaria amoena*	Reduces drug excretion.	[27]
5	Shikonin (**5**)	*Echium plantagineum*	Inhibits the formation of *C. albicans* biofilm; inhibits hyphal formation and adhesion, and enhances the production of farnesol.	[28]
6	Tannins (**6**)	Blueberry, grape, Mangrove *Laguncularia racemosa*	Inhibit the adhesion of *C. albicans*.	[29]
7	α-Mangostin (**7**)	*Garcinia mangostana*		[30]
8	Hexane and ethyl acetate extracts of raspberry	*Rubus idaeus*		[31]
9	Nerol (**8**)	Rutaceous	For the treatment of *C. albicans* invasion.	[32]
10	Pulsatilla decoction	*Pulsatilla chinensis*, *Phellodendri Chinensis Cortex*, *Coptis chinensis*, *Cortex Fraxini*	Inhibits the adhesion of *C. albicans*.	[33]
11	Phloretin (**9**)	Apple peel, pear tree, strawberry	Inhibits the biofilm formation and suppresses the yeast hyphae transition via downregulation genes related to hypha, represses the proteases and phospholipases secretion by reducing the expression of protease-encoding genes *Sap*1 and *Sap*2 as well as PLB1.	[34,35,36]
12	Lepidine B (**10**)	*Lepidium Sativum*	Inhibit the production of phospholipase.	[37]
13	Lepidine E (**11**)			
14	Oil of *Origanum vulgare*			[38]
15	Methanolic extract of *Juglans regia*		Affects *Candida* growth and hydrolytic enzyme secretions.	[39]
16	Acetone and water crude extracts of *Eugenia uniflora*		Affects the transformation from yeast to hyphae and impairs the secretion of phospholipase and proteases.	[40]
17	Pyridoxatin (VB6) (**12**)	Fish, animal liver, legumes, *Lichen endophyte*	Interferes with ergosterol synthesis.	[41]
18	5,6,8-Trihydroxy-7,4′dimethoxyflavone (**13**)	*Dodonaea viscosa* var. *angustifolia*	Inhibits ergosterol synthesis and hyphae and biofilm production in *C. albicans*.	[42]
19	Magnolol (**14**)	*Magnolia garrettii*	Inhibit adhesion and the transition from yeast to hypha and has potential inhibitory effects on *C. albicans*. biofilm formation.	[43]
20	Honokiol (**15**)			
21	Luteolin (**16**)	Perilla, peppermint, *Verbascum lychnitis*	Inhibits adhesion of *C. albicans* and biofilm formation.	[44]
22	Resveratrol (**17**)	Grape, Berry, peanut	Inhibits biofilm formation and disrupts preformed biofilms.	[45]
23	Pterostilbene (**18**)	*Vitis rupestris*, *Pterocarpus marsupium*		
24	Solamargine (**19**)	*Solanum mammosum*	Affects biofilm formation.	[46]
25	Magnoflorine (**20**)	*Acoruscalamus*, *Tinospora cordifolia*	Reduces *C. albicans* biofilm formation.	[47]
26	Propylene glycol extract of *Rosmarinus officinalis*		Has an antibiofilm effect.	[48]
27	Aqueous extract of *Adenophora triphylla* var. *japonica*		Inhibits *Candida* biofilm formation.	[49]
28	Ethanol extract of lemongrass		Reduces *C. albicans* biofilm.	[50]
29	Myricetin (**21**)	Bayberry, *Solanum scabrum*	Interferes with biofilm formation.	[51]
30	Oleuropein (**22**)	*Canarium album*, *Syringa reticulata*	Regulates the morphological transformation of *C. albicans*.	[52]
31	*Paeonia lactiflora* ethanol extract		Inhibits adhesion, morphological transition from pseudohizophae to hyphae, and biofilm formation.	[53]
32	6,7,4′-O-Triacetylxanthin (**23**)	*Scutellaria baicalensis*	In combination with FLZ, inhibits the myceliun and biofilm via Ras/cAMP/PKA signaling pathway.	[54]
33	Tetrandrine (**24**)	*Stephania tetrandra*	Inhibits biofilm formation by decreasing adhesion and morphological transformation. The mechanism of anti-biofilm may be related to the Ras/cAMP pathway.	[55,56]
34	Allicin (**25**)	*Allium sativum*	Suppresses hyphal formation in *C. albicans*.	[57]
35	Piperine (**26**)	Pepper	Regulates the morphological transformation between yeast and mycelium via restrain mycelial extension and converting mycelial phase into yeast form.	[58]
36	*Padma Hepaten*	Amla fruit, belleric myrobalan	Inhibits *C. albicans* biofilm growth and the yeast-to-hypha morphogenic change.	[59]
37	Green tea	*Camellia sinensis*		
38	Decanoic acid (**27**)	Animal fat	Inhibits transformation from yeast to hyphae, adhesion, and biofilm formation of *C. albicans*.	[60]
39	Roemerine (**28**)	Lotus leaf, *Fibraurea recisa*	Inhibits yeast-to-hyphae transition of *C. albicans* and biofilm formation. The antibiofilm mechanism may be in connection with the cAMP pathway.	[61]
40	Morin (**29**)	*Psidium guajava*	Inhibits biofilm formation and production of other virulence factors in *C. albicans* in a concentration-dependent manner.	[62]
41	Biatriosporin D (**30**)	*Biatriospora* spp.	Inhibits adhesion, hyphal morphogenesis, and biofilm formation of *C. albicans*.	[63]
42	Solasodine-3-O-β-D-glucopyranoside (**31**)	*Solanum nigrum*	Inhibits adhesion, morphological transition, and biofilm formation.	[64]

## Data Availability

Not applicable.
